# Elevated RHAMM as a biomarker for predicting diabetic kidney disease in patients with type 2 diabetes

**DOI:** 10.1093/ckj/sfae196

**Published:** 2024-07-03

**Authors:** Bingxue Qi, Yan Lou, Yongyue Zhu, Yang Chen, Shixin Yang, Fanjie Meng, Zhuo Pan, Shuangshuang Liu, Guanchi Yan, Xiaodan Lu, Li-Hao Huang

**Affiliations:** Department of Nephrology, The Second Hospital of Jilin University, Changchun, China; Department of Endocrinology, Jilin Province People's Hospital, Changchun, China; Department of Nephrology, The Second Hospital of Jilin University, Changchun, China; Clinical Medicine College, Changchun University of Chinese Medicine, Changchun, China; Clinical Medicine College, Changchun University of Chinese Medicine, Changchun, China; Department of Endocrinology, Jilin Province People's Hospital, Changchun, China; Department of Endocrinology, Jilin Province People's Hospital, Changchun, China; Precision Molecular Medicine Center, Jilin Province People's Hospital, Changchun, China; Shanghai Key Laboratory of Metabolic Remodeling and Health, Institute of Metabolism and Integrative Biology, Liver Cancer Institute, Zhongshan Hospital, Fudan University, Shanghai, China; Department of Endocrinology, Affiliated Hospital to Changchun University of Chinese Medicine, Changchun University of Chinese Medicine, Changchun, China; Precision Molecular Medicine Center, Jilin Province People's Hospital, Changchun, China; Shanghai Key Laboratory of Metabolic Remodeling and Health, Institute of Metabolism and Integrative Biology, Liver Cancer Institute, Zhongshan Hospital, Fudan University, Shanghai, China

**Keywords:** biomarker, CD44, diabetic kidney disease, hyaluronan, RHAMM, type 2 diabetes

## Abstract

**Background:**

Diabetic kidney disease (DKD) poses a significant challenge globally as a complication of diabetes. Hyaluronan (HA), a critical non-sulfated glycosaminoglycan in the extracellular matrix, plays a pivotal role in the progression of DKD. This study assesses the predictive significance of HA's corresponding receptor, RHAMM (receptor for HA-mediated motility), in DKD pathogenesis in type 2 diabetes (T2DM) patients.

**Methods:**

Enzyme-linked immunosorbent assays were utilized to measure plasma and urine levels of HA, CD44 and RHAMM in 99 diabetic patients. Immunohistochemistry staining was employed to examine HA deposition, CD44 and RHAMM expressions from 18 biopsy-proven DKD patients. Spearman correlation analysis, linear regression and receiver operating characteristic (ROC) analysis were conducted to establish associations between plasma HA, CD44 and RHAMM levels, and clinical parameters in DKD patients with T2DM.

**Results:**

Elevated plasma and urine HA, CD44 and RHAMM levels were notably observed in the severe renal dysfunction group. Plasma RHAMM exhibited positive correlations with HA (r = 0.616, *P* < .001) and CD44 (r = 0.220, *P* < .001), and a negative correlation with estimated glomerular filtration rate (eGFR) (r = –0.618, *P* < .001). After adjusting for other potential predictors, plasma RHAMM emerged as an independent predictor of declining eGFR (β = –0.160, *P* < .05). Increased HA, CD44 and RHAMM levels in kidney biopsies of DKD patients were closely associated with heightened kidney injury. The ROC curve analysis highlighted an area under the curve (AUC) of 0.876 for plasma RHAMM, indicating superior diagnostic efficacy compared to CD44 in predicting DKD pathogenesis. The combined AUC of 0.968 for plasma RHAMM, HA and CD44 also suggested even greater diagnostic potential for DKD pathogenesis.

**Conclusion:**

These findings provide initial evidence that elevated RHAMM levels predict DKD pathogenesis in T2DM patients. The formation of a triple complex involving HA, CD44 and RHAMM on the cell surface shows promise as a targetable biomarker for early intervention to mitigate severe renal dysfunctions.

## Graphical Abstract

**Graphical Abstract:**
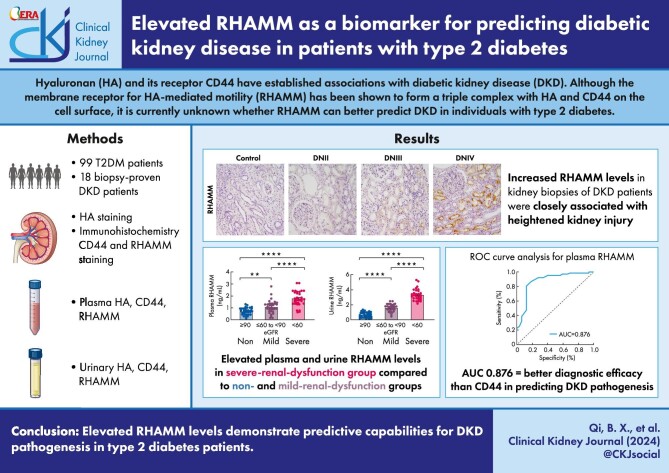

KEY LEARNING POINTS **What was known:**

- Diabetic kidney disease (DKD) is a recognized complication of diabetes, contributing to an increased risk of hypertension and cardiovascular complications.
- Hyaluronan (HA), a crucial non-sulfated glycosaminoglycan in the extracellular matrix, and its receptor CD44 have established associations with DKD.
- Although the membrane receptor for HA-mediated motility (RHAMM) has been shown to form a triple complex with HA and CD44 on the cell surface, it is currently unknown whether RHAMM can better predict DKD in individuals with type 2 diabetes (T2DM).

**This study adds:**

- Elevated plasma RHAMM emerges as an independent predictor for a decline in the estimated glomerular filtration rate.
- The receiver operating characteristics curve analysis reveals superior diagnostic efficacy for DKD pathogenesis with plasma RHAMM compared with CD44.
- Elevated RHAMM levels demonstrate predictive capabilities for DKD pathogenesis in T2DM patients.

**Potential impact:**

- The study suggests that a triple complex formation involving RHAMM, HA and CD44 on the cell surface could serve as a promising targetable biomarker for early intervention in mitigating severe renal dysfunctions.

## INTRODUCTION

The surge in diabetes cases significantly heightens the risk of complications, notably chronic kidney disease (CKD) and end-stage kidney disease (ESKD), which contributes significantly to reduced lifespan among individuals with diabetes [1]. In the USA, up to 44% of ESKD cases are linked to diabetes [2]. Moreover, diabetic kidney disease (DKD) substantially elevates the risk of hypertension and cardiovascular issues, imposing considerable economic burdens on individuals, families and society at large. Despite adopting dialysis, the 5-year survival rate for those diagnosed with ESKD is merely 42% [3]. These results underscore the pressing need for new biomarkers to precisely indicate various stages of DKD or identify potential candidates for interventions aimed at preventing DKD.

Hyaluronan (HA), a critical component of the extracellular matrix, regulates inflammation, tissue repair and disease progression [4–7]. Its receptor, CD44, expressed in multiple tissues, including the kidney, plays a pivotal role in diabetic nephropathy [8–10]. Studies show increased CD44 expression in the glomerulus of rats with diabetic nephropathy, significantly correlating with proteinuria [10]. In aged CD44 wild-type mice, heightened CD44 expression triggers phenotypic shifts in parietal epithelial cells, leading to glomerular hypertrophy, reduced podocyte density and glomerular sclerosis [11]. Notably, a CD44 signature has demonstrated efficacy in distinguishing between DKD and diabetes [12].

Another crucial HA receptor, the receptor for HA-mediated motility (RHAMM or CD168), though structurally distinct from CD44, shares similar functionalities in various diseases, such as inflammation, angiogenesis [13, 14], tissue response to injury [15, 16] and tumorigenesis [17, 18]. While RHAMM mRNA is usually challenging to detect in steady-state tissues, it remains persistently elevated in damaged tissues or disease states [19]. In excisional skin wounds, a RHAMM mimetic peptide capable of blocking HA signaling has shown efficacy in reducing inflammation and fibrogenesis [20], demonstrating the pivotal role of RHAMM during inflammation. Although RHAMM's role in diseases has been studied akin to CD44 [18, 21], no studies to date have reported on the relationship between RHAMM and DKD. The potential integral involvement of RHAMM with HA and CD44 in the DKD development also remains unclear.

This present study aims to evaluate HA, CD44 and RHAMM levels in plasma, urine and kidney biopsies of diabetic patients with and without DKD. The primary objective is to determine whether RHAMM can emerge as a precise and novel biomarker for DKD pathogenesis.

## MATERIALS AND METHODS

### Patients

A total of 99 participants were recruited from the Department of Endocrinology at Jilin Province People's Hospital between February 2023 and October 2023. The inclusion criteria encompassed individuals aged 18 to 75 years who were newly or previously diagnosed with type 2 diabetes mellitus (T2DM) according to the World Health Organization's Diagnostic Criteria for T2DM in 1999 [22]. Participants with DKD were identified based on a persistent urinary albumin-to-creatinine ratio (UACR) exceeding 30 mg/g and a progressive reduction in estimated glomerular filtration rate (eGFR) to ≤60 mL/min/1.73 m^2^ [23]. Exclusion criteria comprised individuals with acute kidney injury from various causes, pregnant or lactating women, patients with malignancies, cardiovascular or respiratory diseases, blood system disorders, immune system diseases, primary kidney diseases or hypertension, as well as those with severe mental illness or an inability to cooperate with researchers. We employed two criteria, based on eGFR levels and albuminuria, to stratify patients into distinct categories for investigating the roles of HA and its receptors CD44 and RHAMM in diabetic-associated kidney injury. Initially, we categorized participants based on the severity of CKD using eGFR levels, with lower eGFR levels indicative of greater clinical complications [23]. This resulted in the classification of participants into three groups: the non-renal dysfunction group (NRG: eGFR ≥90 mL/min/1.73 m^2^, *n* = 34), the mild renal dysfunction group (MRG: eGFR: 60–90 mL/min/1.73 m^2^, *n* = 34) and the severe renal dysfunction group (SRG: eGFR <60 mL/min/1.73 m^2^, *n* = 31) (Table 1). Additionally, to provide further insight, we categorized participants into three albuminuria categories, a commonly utilized approach for initial disease assessment and prognostication due to its simplicity. This resulted in the classification of participants in to three groups: the normal control group (NCG: UACR <30 mg/g, *n* = 33), the microalbuminuria group (MIG: UACR 30–300 mg/g, *n* = 31) and the macroalbuminuria group (MAG: UACR ≥300 mg/g, *n* = 35) (Supplementary data, Table S1).

**Table 1:** Comparison of clinical parameters between three groups.

Variables	NRG (eGFR ≥90)	MRG (60 ≤ eGFR < 90)	SRG (eGFR <60)	*P*
*n* (man/woman)	34 (17/17)	34 (16/18)	31 (16/15)	
Age (years)	55.18 ± 2.09	60.59 ± 1.68	60.29 ± 2.69	.1383
Clinical course (years)	10.24 ± 1.18	8.88 ± 1.01	20.61 ± 1.16*,**	<.0001
SBP (mmHg)	125.70 ± 1.61	123.60 ± 1.45	147.20 ± 3.81*,**	<.0001
DBP (mmHg)	87.06 ± 1.70	84.79 ± 2.43	81.84 ± 2.06	.2188
BMI (kg/m^2^)	26.39 ± 0.71	25.12 ± 0.61	26.34 ± 0.86	.3737
TG (mmol/L)	3.05 ± 0.41	2.72 ± 0.38	4.25 ± 0.50**	.0380
TC (mmol/L)	4.86 ± 0.23	5.20 ± 0.27	5.17 ± 0.37	.6310
LDL-c (mmol/L)	2.32 ± 0.14	2.40 ± 0.14	2.53 ± 0.25	.7222
HDL-c (mmol/L)	1.19 ± 0.06	1.29 ± 0.06	1.21 ± 0.07	.5258
HbA1c (%)	8.84 ± 0.41	8.15 ± 0.48	8.11 ± 0.51	.4514
FBG (mmol/L)	8.87 ± 0.47	9.71 ± 0.75	9.56 ± 0.79	.6442
PBG (mmol/L)	13.32 ± 0.90	13.21 ± 0.99	15.49 ± 1.58	.3273
Cr (μmol/L)	63.54 ± 2.22	76.85 ± 3.12*	215.10 ± 32.02*,**	<.0001
BUN (μmol/L)	5.31 ± 0.25	6.17 ± 0.27	17.25 ± 1.22*,**	<.0001
α1-microglobulin (u/L)	27.29 ± 1.22	27.94 ± 1.38	55.51 ± 4.25*,**	<.0001
β2-microglobulin (u/L)	2.11 ± 0.10	2.60 ± 0.17	7.79 ± 1.32*,**	<.0001
eGFR (mL/min/1.73 m^2^)	117.8 ± 2.81	81.11 ± 1.13*	38.81 ± 2.76*,**	<.0001

Results were expressed as mean and standard error of the mean.

^*^ *P *< .0001 vs NRG, ^**^P < .0001 vs MRG.

Patients with biopsy-proven diabetic nephropathy (DN) at Stages II, III and IV of Tervaert's renal pathological classification were enrolled, with kidney samples obtained from the Department of Nephrology at the Second Hospital Affiliated to Jilin University, Changchun, China. Kidney samples from controls were sourced from the Urinary Surgery Department, comprising normal kidney tissues from diabetic patients undergoing kidney operations due to renal cysts without other renal diseases. Participants were divided into the normal control group (Ctrl: renal biopsy with diabetes, *n* = 3), the DN Stage II group (DN II: renal biopsy proved DN at Stage II of Tervaert's renal pathological classification, *n* = 5), the DN Stage III group (DN III: renal biopsy proved DN at Stage III of Tervaert's renal pathological Classification, *n* = 5) and the DN Stage IV group (DN IV: renal biopsy proved DN at Stage IV of Tervaert's renal pathological Classification, *n* = 5).

All participants provided informed consent in accordance with the Declaration of Helsinki of the World Medical Association. The study received approval from the Research Ethics Committee of Jilin Province People's Hospital after obtaining informed consent from the patients.

### Measurement of clinical parameters

Clinical parameters of these patients were collected from the enrolled patients, encompassing demographic information, including age, sex, clinical course, systolic blood pressure (SBP), diastolic blood pressure (DBP) and body mass index (BMI). Venous blood sampling was carried out in the early morning after overnight fasting. Fasting blood glucose (FBG), postprandial blood glucose (PBG), hemoglobin A1c (HbA1c), triglyceride (TG), cholesterol (TC), high-density lipoprotein-cholesterol (HDL-c), low-density lipoprotein-cholesterol (LDL-c), serum creatinine (Cr), blood urea nitrogen (BUN), α1-microglobulin, β2-microglobulin, urinary albumin-to-creatinine ratio (UACR) and eGFR levels were measured at the Department of Clinical Medicine, Jilin Province People's Hospital. The eGFR was calculated using Chronic Kidney Disease Epidemiology Collaboration formula [24]. Plasma was isolated and processed according to the standard protocol and stored at –80°C for subsequent biomarkers assay, which was conducted all at once across the entire sample group at a later date.

### Measurement of circulating concentration of HA, CD44 and RHAMM

Concentrations of plasma and urine HA, CD44 and RHAMM were measured with enzyme-linked immunosorbent assay (ELISA) kit, according to protocols provided by the manufacturer [Human Cluster of Differentiation (CD44) ELISA Kit; lot no. CSB-E11846h; CUSABIO, Wuhan, China; Human Hyaluronic acid (HA) ELISA Kit; lot no. CSB-E04805h; CUSABIO, Wuhan, China; and Human Hyaluronan Mediated Motility Receptor (HMMR) ELISA Kit, lot no. CSB-E04805h; Cloud-Clone Corp, Wuhan, China].

### Immunohistochemistry

Immunohistochemistry staining of paraffin-embedded kidney sections was performed to determine the expression levels of CD44 and RHAMM using anti-CD44 (#E7K2Y, Cell Signaling, 1:200) and anti-RHAMM (#PV-8000-1, ORIGENE, 1:200), horseradish peroxidase–linked anti-rabbit (#7074S, Cell Signaling, 1:1000) and DAB Substrate kit (#D3939, Sigma). HA was assessed using a biotinylated HA-binding protein (#AMS.HKD-BC41, amsbio, 1:200). Images were captured using a camera mounted on an AxioVision microscope. Quantifications of HA, CD44 and RHAMM staining were assessed in the cortex area using ImageJ software.

### Statistical analysis

Data were expressed as mean ± standard error of the mean and analyzed using GraphPad Prism (Version 9) and SPSS (Version 26.0). Comparisons among multiple groups were performed using one-way analysis of variance (ANOVA) followed by Tukey's multiple comparisons test. Spearman correlation analysis was used to evaluate the correlation between plasma HA, CD44 and RHAMM, and clinical parameters. Simple linear regression was used to evaluate the correlation between eGFR and plasma HA, CD44 and RHAMM as well as clinical parameters, and the correlation between UACR and urine HA, CD44 and RHAMM. Risk factors for decreasing eGFR based on plasma HA, CD44 and RHAMM were obtained using multiple linear regression models. The diagnostic efficiency was evaluated by receiver operating characteristic (ROC) curve. The data were considered statistically significant at *P* < .05.

## RESULTS

### Participant characteristics

A total of 99 patients with T2DM were included, comprising 49 men and 50 women. The distribution across the groups included 34 patients in NRG with an eGFR ≥90 mL/min/1.73 m^2^, 34 patients in MRG with an eGFR ranging from 60 to 90 mL/min/1.73m^2^, and 31 patients in SRG with eGFR <60 mL/min/1.73 m^2^. A comparison of clinical parameters showed that age, DBP, BMI, TG, TC, LDL-c, HDL-c, HbA1c, FBG and PBG between the three groups were not significantly different. However, clinical course and SBP were higher in diabetic patients in SRG compared with the NRG and MRG (*P *< .0001). Serum Cr in MRG was higher than that in NRG; eGFR in MRG was lower than that in NRG; whereas no difference was observed for blood urea nitrogen (BUN), α1-macroglobulin and β2-macroglobulin between NRG and MRG. However, these clinical parameters were higher in SRG compared with NRG and MRG (*P *< .0001) (Table 1).

### Plasma and urine HA, CD44 and RHAMM levels are significantly higher in SRG than in MRG or NRG

**Figure 1:**
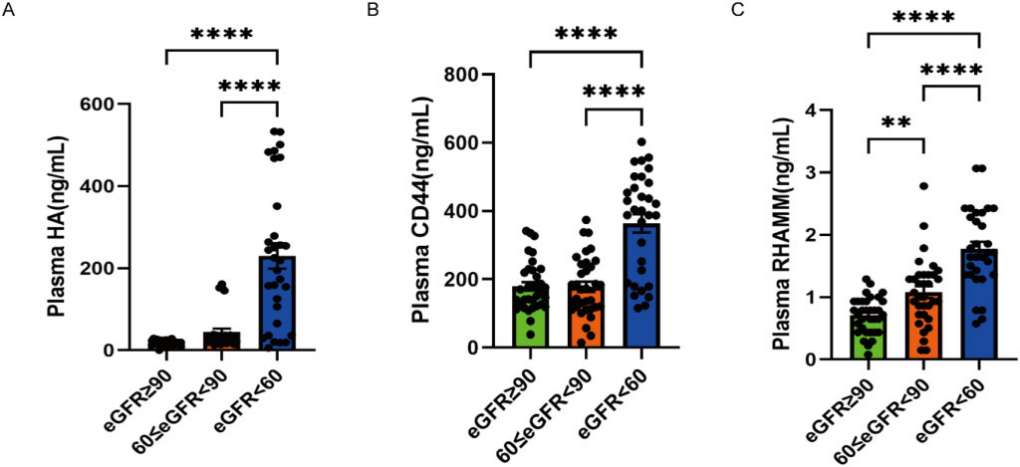
Plasma HA, CD44 and RHAMM levels. Plasma HA, CD44 and RHAMM levels were measured by ELISA from 99 diabetic patients with or without DKD. Comparisons among NRG (eGFR ≥90 mL/min/1.73 m^2^), MRG (60 ≤ eGFR <90 mL/min/1.73 m^2^) and SRG (eGFR <60 mL/min/1.73 m^2^) groups were analyzed using one-way ANOVA followed by Tukey's multiple comparison test. Plasma HA (**A**) was significantly higher in SRG than in NRG and MRG; plasma CD44 (**B**) was significantly higher in SRG than in NRG and MRG; plasma RHAMM (**C**) was significantly higher in MRG than in NRG and significantly higher in SRG than in NRG and MRG. ^∗∗^*P* < .01 and ^∗∗∗∗^*P* < .0001.

**Figure 2:**
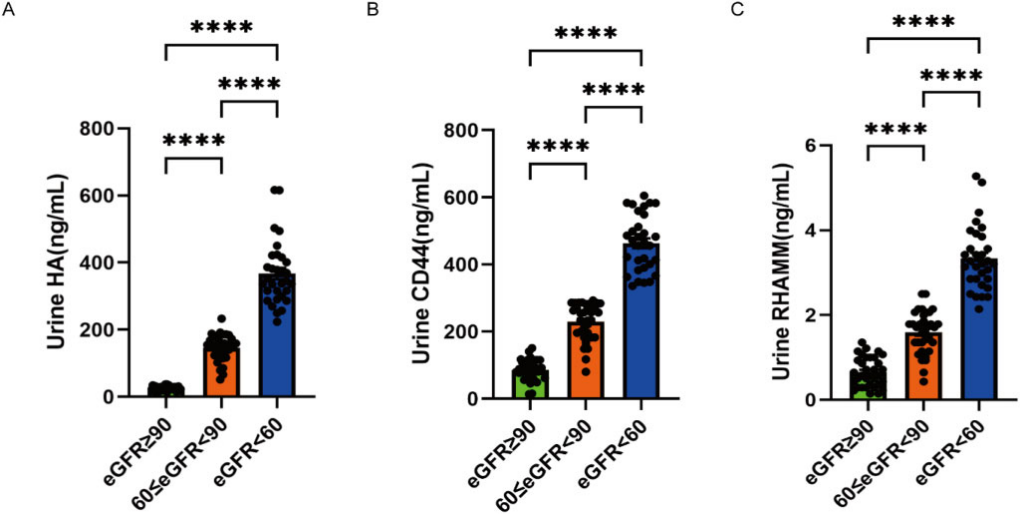
Urine HA, CD44 and RHAMM levels. Urine HA, CD44 and RHAMM levels were measured by ELISA from 99 diabetic patients with or without DKD. Comparisons among NRG (eGFR ≥90 mL/min/1.73 m^2^), MRG (60 ≤ eGFR <90 mL/min/1.73 m^2^) and SRG (eGFR <60 mL/min/1.73 m^2^) groups were analyzed using one-way ANOVA followed by Tukey's multiple comparison test. Urine HA (**A**) and CD44 (**B**) as well as RHAMM (**C**) were significantly higher in MRG than in NRG and significantly higher in SRG than in NRG and MRG. ^∗∗∗∗^*P* < .0001.

ELISA was employed to evaluate plasma and urine HA, CD44 and RHAMM levels in 99 patients. Comparing SRG (eGFR <60 mL/min/1.73 m^2^) with both NRG (eGFR ≥90 mL/min/1.73 m^2^) and MRG (60 ≤ eGFR < 90 mL/min/1.73 m^2^), notable increases were observed in plasma and urine levels of HA, CD44 and RHAMM (Figs 1 and 2). Specifically, RHAMM levels in plasma were significantly higher in MRG compared with NRG (Fig. 1C); in addition, levels of HA, CD44 and RHAMM in urine were all notably elevated in MRG compared with NRG (Fig. 2A–C).

### The correlation between plasma HA, CD44 and RHAMM levels, and clinical parameters

**Figure 3:**
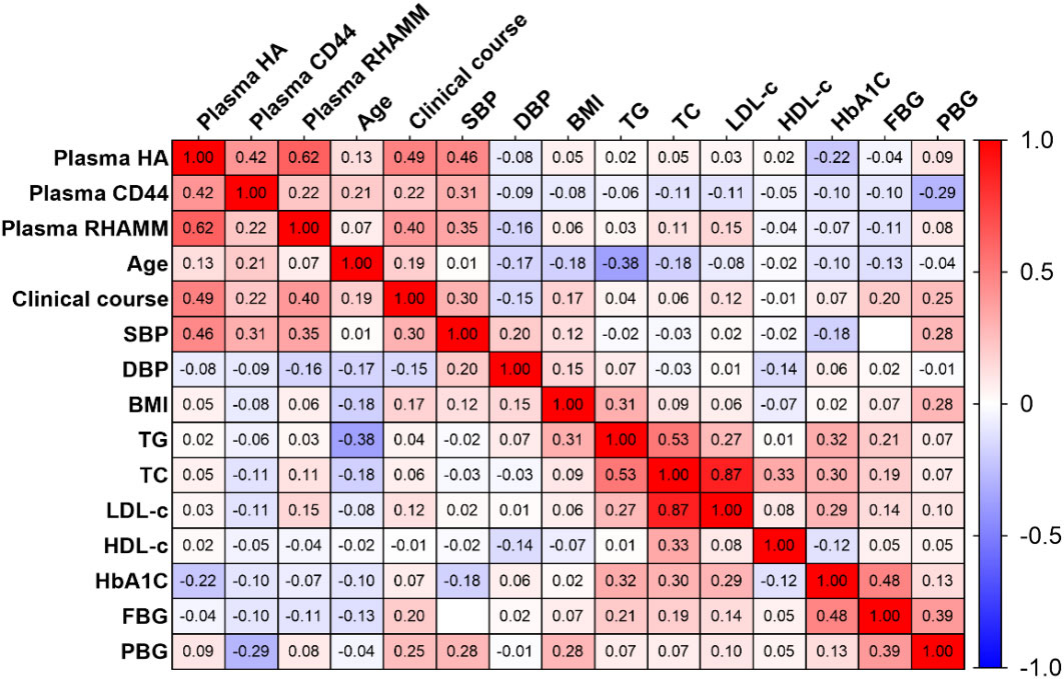
The correlation between plasma HA, CD44 and RHAMM levels and clinical parameters. Spearman correlation analysis was used to evaluate the correlation between plasma HA, CD44 and RHAMM and clinical parameters. Plasma HA, CD44 and RHAMM positively correlated with each other. Plasma HA was positively correlated with clinical course and SBP, and negatively correlated with HbA1c. Plasma CD44 was positively correlated with age, clinical course and SBP; a negative correlation was seen in PBG. Plasma RHAMM was positively correlated with both course and SBP.

Given the significant elevation of plasma levels of HA, CD44 and RHAMM in SRG (Fig. 1), we proceeded to assess their potential associations with each other and with additional clinical parameters using Spearman correlation analysis. Among the 99 patients in Study 1, the analysis revealed that plasma HA exhibited a positive correlation with both plasma CD44 (r = 0.415, *P* < .001) and RHAMM (r = 0.616, *P* < .001). Additionally, a positive correlation was observed between plasma CD44 and RHAMM (r = 0.220, *P* < .05). Further correlations were identified between these markers and clinical parameters. Plasma HA showed positive correlations with clinical course (r = 0.489, *P* < .001) and SBP (r = 0.464, *P* < .001), while exhibiting a negative correlation with HbA1c (r = –0.219, *P* < .05). Plasma CD44 demonstrated positive correlations with age (r = 0.206, *P* < .05), clinical course (r = 0.219, *P* < 0.05) and SBP (r = 0.311, *P* < .05), with a negative correlation observed in PBG (r = –0.287, *P* < .05). Plasma RHAMM exhibited positive correlations with both clinical course (r = 0.398, *P* < .0001) and SBP (r = 0.352, *P* < .001) (Fig. 3). These findings suggest a positive association among plasma HA, CD44 and RHAMM. Interestingly, they are all positively correlated with the clinical course, indicating that these molecules may serve as potential biomarkers for DKD pathogenesis.

### The correlation between plasma HA, CD44 and RHAMM levels and DKD

**Figure 4:**
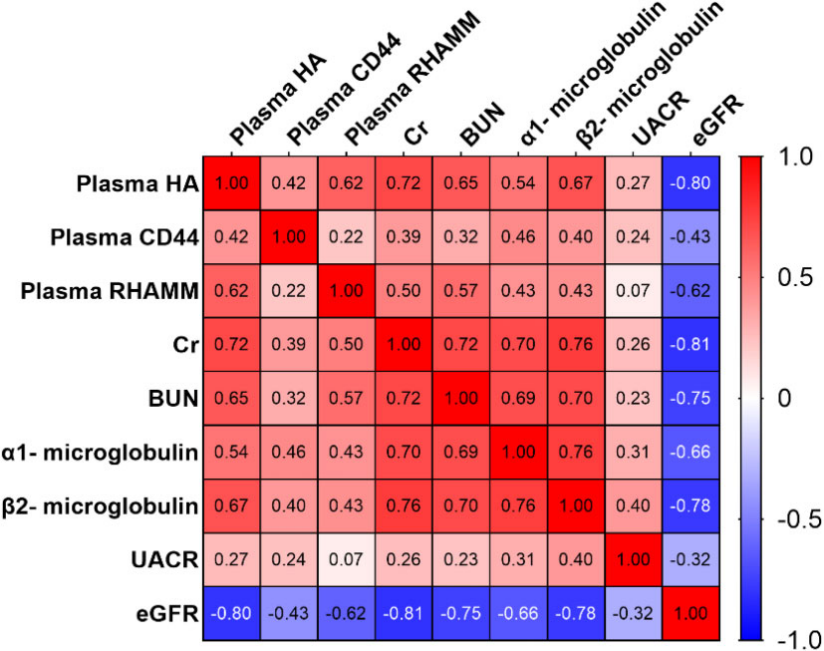
The correlation between plasma HA, CD44 and RHAMM levels and DKD. Spearman correlation analysis was performed to evaluate the correlation between plasma HA, CD44 and RHAMM levels and DKD.

In order to delve deeper into the association between plasma HA, CD44 and RHAMM levels, and DKD, Spearman correlation analysis was performed. Among the 99 patients, plasma HA exhibited positive correlations with Cr (r = 0.716, *P* < .001), BUN (r = 0.653, *P* < .001), α1-microglobulin (r = 0.539, *P* < .001), β2-microglobulin (r = 0.675, *P* < .001) and UACR (r = 0.267, *P* < .01), and a negative correlation with eGFR (r = –0.798, *P* < .001). Similarly, plasma CD44 showed positive correlations with serum Cr (r = 0.392, *P* < .001), BUN (r = 0.325, *P* < .001), α1-microglobulin (r = 0.457, *P* < .001), β2-microglobulin (r = 0.399, *P* < .001) and UACR (r = 0.244, *P* < .05), and a negative correlation with eGFR (r = –0.434, *P* < .001). Additionally, plasma RHAMM exhibited positive correlations with serum Cr (r = 0.502, *P* < .001), BUN (r = 0.570, *P* < .001), α1-microglobulin (r = 0.426, *P* < .001) and β2-microglobulin (r = 0.435, *P* < .001), and a negative correlation with eGFR (r = –0.618, *P* < .001) (Fig. 4). These findings highlight that plasma HA, CD44 and RHAMM levels are not only positively correlated with various indicators of kidney function but are also negatively correlated with the eGFR, suggesting that these molecules have the potential to predict DKD pathogenesis.

### Immunohistochemical expression of HA, CD44 and RHAMM levels in human kidney biopsies

**Figure 5:**
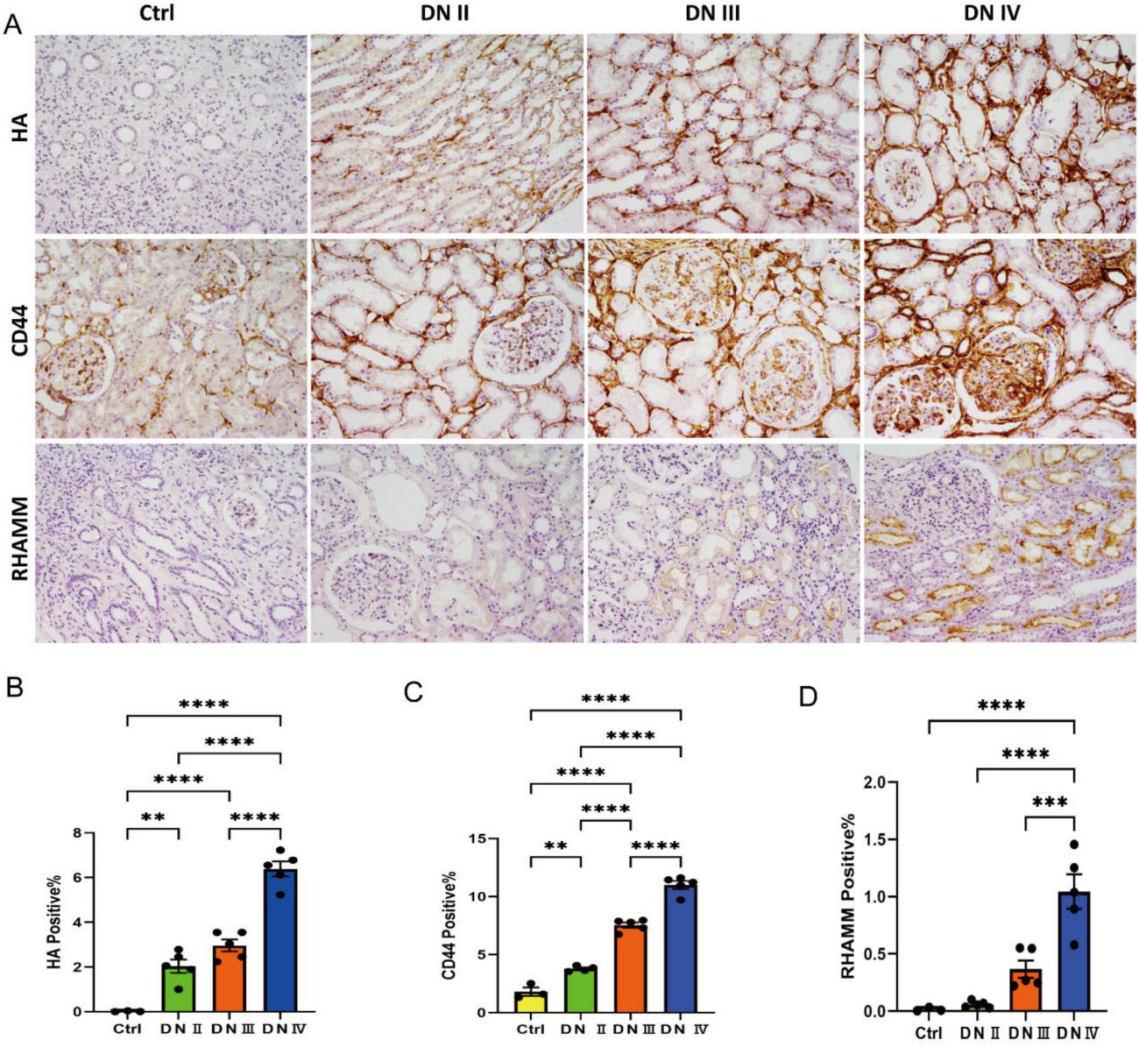
The levels of HA, CD44 and RHAMM in kidney biopsies. (**A**) CD44 was mostly localized on the mesangial area of the glomerulus and basolateral membranes of the nephric tubule in the inner stripes of the outer medulla, and HA (**B**) and CD44 (**C**) expression was gradually increased in the kidneys at various stages of DN in patients. RHAMM (A) was mostly localized on the surface of proximal curved tubule parietal epithelial cells, and the protein expression level of RHAMM (**D**) was significantly higher in DN Ⅳ group than Ctrl, DN Ⅱ and DN Ⅲ group. ^∗∗^*P* < .01, ^∗∗∗^*P* < .001 and ^∗∗∗∗^*P* < .0001.

To further elucidate the location and expression patterns of HA, CD44 and RHAMM in kidney biopsies, the immunohistochemical analysis was performed on a total of 18 renal biopsy specimens obtained from patients with biopsy-proven DN at various stages (stages II, III and IV of Tervaert's renal pathological classification). The analysis revealed that CD44 predominantly localized in the mesangial area of the glomerulus and on the basolateral membranes of the nephric tubule in the inner stripes of the outer medulla. Notably, the expression levels of HA and CD44 increased gradually with the progression of DN stages (Fig. 5A–C). Furthermore, the expression of RHAMM was evaluated across different stages of DN. The results indicated that RHAMM primarily was localized on the surface of proximal curved tubule parietal epithelial cells, with significantly higher expression levels observed in the DN IV group compared with the Ctrl, DN II and DN III groups (Fig. 5A and D). These findings suggest a correlation between kidney injury and increased expression of HA, CD44 and RHAMM in kidney biopsies in patients with DKD.

### The association of eGFR and UACR with plasma or urine HA, CD44 and RHAMM levels, and clinical parameters

**Figure 6:**
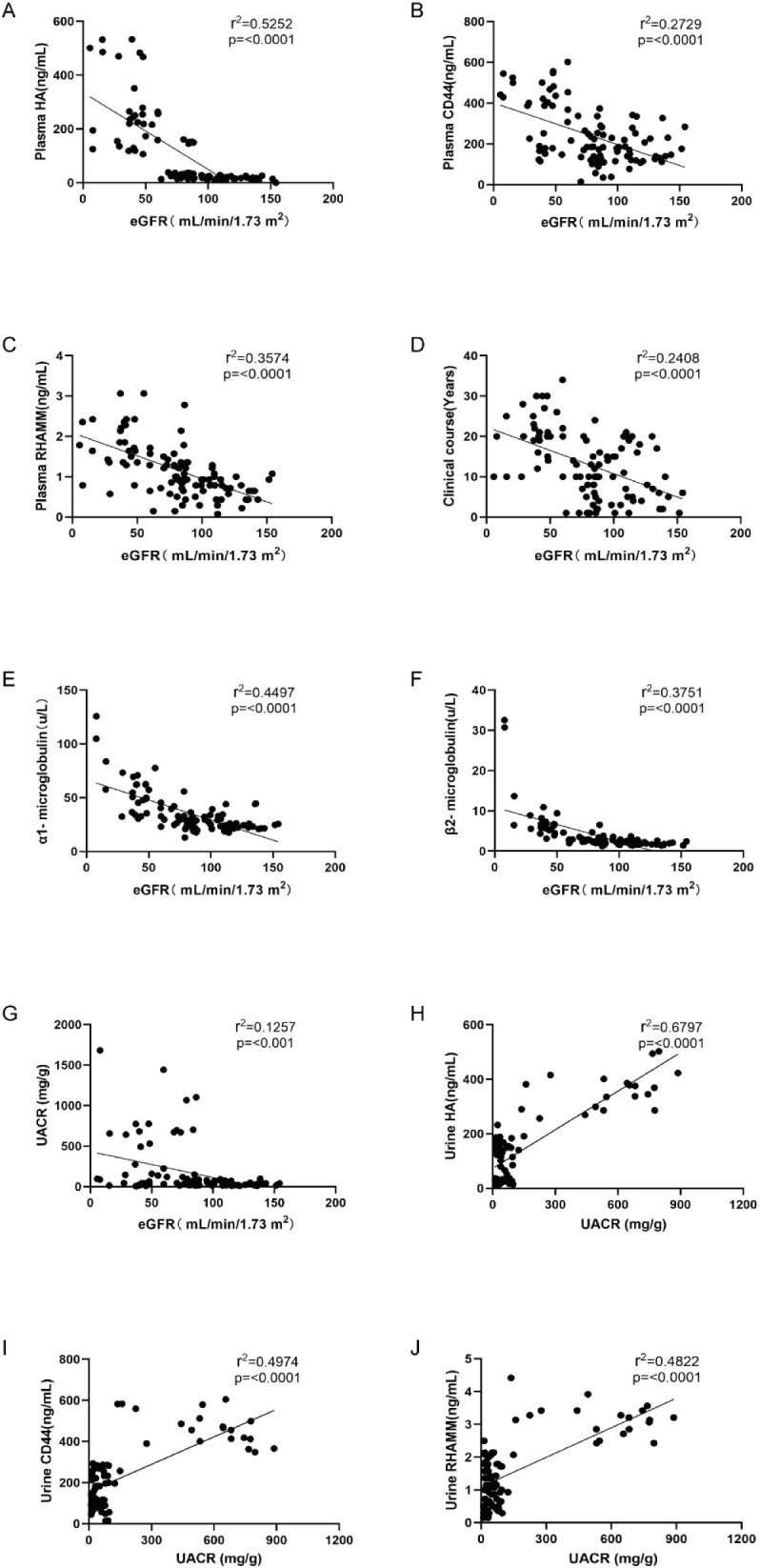
The association of eGFR and UACR with plasma ((A) to (F)) or urine ((G) to (J)) HA, CD44 and RHAMM levels as well as with clinical parameters. Simple linear regression was performed to evaluate the correlation between eGFR and UACR and plasma or urine HA, CD44 and RHAMM levels, and clinical parameters. Pearson R^2^ and *P*-values are given on the graph. Individual data points are shown with a line of best fit (solid).

Based on the findings from Figs 4 and 5, it is evident that both plasma and kidney biopsies of HA, CD44 and RHAMM levels were associated with DKD pathogenesis. A simple linear regression analysis was conducted to understand these associations further and evaluate the relationship between plasma HA, CD44 and RHAMM levels, and eGFR. The eGFR serves as a comprehensive measurement of the total filtration rates of functioning nephrons in the kidney and is an optimal metric for assessing kidney function and associated with clinical complications [23]. The results revealed significant and negative associations between eGFR levels and plasma HA (r^2 ^= 0.5252, *P* < .0001) (Fig. 6A), CD44 (r^2 ^= 0.2729, *P* < .0001) (Fig. 6B) and RHAMM (r^2 ^= 0.3574, *P* < .0001) (Fig. 6C). Additionally, clinical course (r^2 ^= 0.2408, *P* < .0001) (Fig. 6D), SBP (r^2 ^= 0.3274, *P* < .0001) (Supplementary data, Fig. S1A), serum Cr (r^2 ^= 0.4395, *P* < .0001) (Supplementary data, Fig. S1B), BUN (r^2 ^= 0.5462, *P* < .0001) (Supplementary data, Fig. S1C), α1-macroglobulin (r^2 ^= 0.4497, *P* < 0.0001) (Fig. 6E), β2-microglobulin (r^2 ^= 0.3751, *P* < .0001) (Fig. 6F) and UACR (r^2 ^= 0.1257, *P* < .001) (Fig. 6G) were also significantly and negatively associated with eGFR levels. Furthermore, an assessment of the association between eGFR levels and BMI, FBP, PBG, HbA1c, TC, TG, HDL-c and LDL-c (Supplementary data, Fig. S1). These findings collectively demonstrate a negative correlation between eGFR levels and plasma HA, CD44 and RHAMM levels.

Next, to further evaluate whether urine HA, CD44 and RHAMM levels exhibit associations with albuminuria levels, we categorized patients into three albuminuria categories based on UACR (Supplementary data, Table S1). Subsequently, we investigated the relationship between urine levels of HA, CD44 and RHAMM, and UACR. The results revealed significant and positive associations between UACR levels and urine HA (r^2 ^= 0.6797, *P* < .0001) (Fig. 6H), CD44 (r^2 ^= 0.4974, *P* < .0001) (Fig. 6I) and RHAMM (r^2 ^= 0.4822, *P* < .0001) (Fig. 6J), showing that higher urine HA, CD44 and RHAMM levels indicate higher albuminuria and disease severity. These results are consistent with the negative associations observed between plasma HA, CD44 and RHAMM, and eGFR, as illustrated in Fig. 6A–C, underscoring the potential of these biomarkers in indicating kidney disease severity.

### The analysis of potential influencing factors to eGFR

In light of the potential role of plasma HA, CD44 and RHAMM levels as biomarkers for predicting DKD, we conducted an analysis to determine whether these biomolecules and other clinical parameters could function as independent risk factors for eGFR. Multiple linear regression analysis was employed, with eGFR levels designated as the dependent variable and plasma HA, CD44 and RHAMM levels, as well as additional clinical parameters (such as clinical course, SBP, Cr, BUN, α1-macroglobulin, β2-microglobulin and UACR) serving as independent variables. The results revealed the overall significance of the multiple regression equation (F = 22.272, *P* < .01). Notably, plasma RHAMM (β = –0.160, *P* < .05), serum Cr (β = –0.285, *P* < .01) and UACR (β = –0.158, *P* < .05) were identified as statistically significant contributors, signifying that plasma RHAMM (B < 0, *P* < .05), serum Cr (B < 0, *P* < .01) and UACR (B < 0, *P* < .05) are independent risk factors associated with a decline in eGFR (Table 2). These findings underscore the potential clinical relevance of plasma RHAMM, serum Cr and UACR as independent indicators for predicting a decrease in eGFR in individuals with DKD.

**Table 2:** Multiple linear regression analysis for eGFR levels in patients with DKD.

Variables	B	SE	Beta	*t*	*P*
Constants	148.659	19.388		7.667	.000
Plasma HA	–0.058	0.031	–0.228	–1.873	.065
Plasma CD44	–0.009	0.022	–0.037	–0.427	.671
Plasma RHAMM	–8.145	3.964	–0.160	–2.055	**.043**
Course	–0.460	0.327	–0.113	–1.407	.163
SBP	–0.215	0.158	–0.107	–1.363	.177
Cr	–0.092	0.033	–0.285	–2.789	**.007**
BUN	–0.709	0.652	–0.139	–1.088	.280
α1-macroglobulin	0.110	0.235	0.061	0.469	.640
β2-microglobulin	0.031	0.018	0.102	1.765	.081
UACR	–0.017	0.008	–0.158	–2.192	**.031**

Significant values are highlighted in bold.

### Diagnostic efficacy of plasma HA, CD44 and RHAMM in DKD

**Figure 7:**
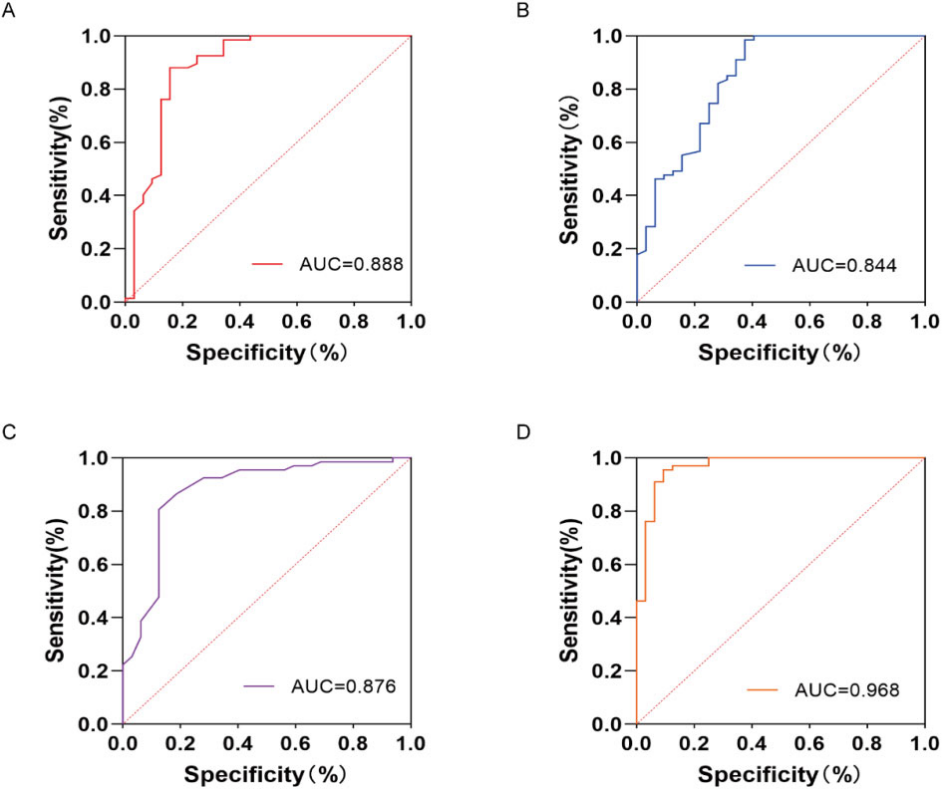
Diagnostic efficacy of plasma HA, CD44 and RHAMM in DKD. Risk factors for decreasing eGFR based on plasma HA, CD44 and RHAMM levels were obtained using multiple linear regression models. The diagnostic efficiency was evaluated by ROC curve analysis. The ROC curve to diagnose DKD revealed an AUC of 0.888 for HA (**A**), 0.844 for CD44 (**B**). 0.876 for RHAMM (**C**), and 0.968 for HA, CD44 and RHAMM comprehensively (**D**).

The ROC curve analysis of plasma HA, CD44 and RHAMM levels in the DKD pathogenesis showed that plasma HA, CD44 and RHAMM could be used to evaluate DKD pathogenesis. The ROC curve to diagnose DKD revealed an AUC of 0.888 for HA (Fig. 7A), 0.844 for CD44 (Fig. 7B) and 0.876 for RHAMM (Fig. 7C), and 0.968 for HA, CD44 and RHAMM comprehensively (Fig. 7D).

## DISCUSSION

DKD represents a formidable complication of diabetes, hallmarked by glomerulosclerosis. Its progression from latent symptoms to proteinuria, edema and hypertension heightens cardiovascular risks and often culminates in ESKD, a primary cause of mortality in diabetic patients [25]. This underlines an urgent need for precise diagnostic biomarkers to aid disease management and enable early intervention to prevent severe renal dysfunctions [26, 27].

In our current study, we unveiled for the first time that heightened plasma RHAMM is an independent predictor of declining eGFR. ROC curve analysis showed an AUC of 0.876 for plasma RHAMM (Fig. 7C) in predicting DKD pathogenesis, surpassing CD44’s AUC of 0.844 (Fig. 7B). Fascinatingly, an AUC of 0.968 for plasma RHAMM, HA and CD44 signifies even superior diagnostic efficacy for DKD pathogenesis, suggesting that a cell surface triple complex comprising HA, CD44 and RHAMM could potentially serve as a promising targetable biomarker for early intervention in severe renal dysfunctions.

Our investigation also delved into the role of HA in DKD, known to accumulate in diabetic kidney [28], contributing to heightened kidney inflammation and interstitial fibrosis [29, 30]. Notably, inhibition of HA synthesis via 4-MU normalized kidney functions, mesangial expansion score and glomerular injury index in diabetic mice, emphasizing HA's involvement in DKD progression [28]. The association between total HA and low molecular weight HA in the kidney with increased UACR in diabetic type 2 model mice further underscores the link between HA and DKD progression [28]. Our findings indicate elevated HA plasma and urine levels in patients in SRG compared with individuals in NRG and MRG (Figs 1 and 2). Furthermore, the expression of HA in kidney biopsy samples is notably higher in DN IV patients compared with those in the Ctrl, DN II and DN Ⅲ groups (Fig. 5). Our findings align with the existing evidence, emphasizing the potential role of HA in the progression of DKD.

The role of CD44 as the receptor for HA has established its association with DKD pathogenesis. Human and mouse models of focal segmental glomerulosclerosis and diabetic nephropathy have demonstrated CD44-dependent expressions of extracellular matrix protein isoforms in parietal epithelial cells [31]. Moreover, CD44 mRNA expression shows a negative correlation with the GFR in DKD patients [32], and increased levels are evident in renal fibrosis tissue and urine, alongside elevated complement components in DKD [12]. This CD44 signature effectively distinguishes between DKD and diabetes. In line with these findings, our study highlights elevated kidney, urine and plasma CD44 protein expressions in patients with SRG compared with those in NRG and MRG, emphasizing the pivotal role of CD44 in DKD pathogenesis.

RHAMM, functioning as an additional HA receptor, was initially identified as a soluble protein secreted by migrating cells, facilitating cell movement and infiltration. Its extracellular form binds to CD44 on mesenchymal cells in wounds, orchestrating cellular transformation and migration in an HA-dependent manner [15]. Research has indicated the formation of a triple complex involving HA, CD44 and RHAMM on the cell surface, which becomes upregulated in various cell lines following interaction with immobilized HA. This interaction influences the expression of both CD44 and RHAMM, hinting at cell-specific feedback loops within the signaling cascade [33]. Throughout fibrosarcoma progression, the involvement of HA, CD44 and RHAMM in signaling mechanisms significantly impacts essential cellular processes like migration, adhesion and proliferation, potentially leading to fibroblastoid cell malignant transformation [34]. In our current study, notably elevated HA, CD44 and RHAMM protein levels were observed in plasma, urine and kidney biopsies of patients with SRG. The expression levels of these three molecules positively correlate with markers of kidney dysfunction (α1-microglobulin, β2-microglobulin, UACR) and exhibit a negative correlation with eGFR (Fig. 6). This suggests that increased cell surface expressions of HA, CD44 and RHAMM complexes might contribute to kidney injury and dysfunction.

Among these molecules, while RHAMM's involvement in tumorigenesis has been investigated extensively [35–37], no existing research currently details the relationship between RHAMM and DKD. Significantly, plasma RHAMM emerged as a notable independent predictor of declining eGFR in individuals with diabetic kidney disease (Table 2). Diagnostic efficiency analysis illustrated that plasma RHAMM levels surpassed CD44 in predicting DKD, as revealed by ROC curve analysis (Fig. 7). These findings implied that RHAMM might play a role in CD44-mediated inflammation within DKD pathogenesis. However, the combined analysis of all three biomarkers demonstrated the highest sensitivity in predicting DKD (Fig. 7D), underscoring their potential as a collective triple complex for detecting DKD. Although plasma HA, CD44 and RHAMM expressions were elevated in kidney tissue biopsies, further investigation is needed to ascertain whether these potential biomarkers can be utilized for early intervention in DKD.

The study acknowledges several limitations. It is a single-center cross-sectional study, and future research gathering samples from multiple centers could provide a broader perspective, minimizing potential regional bias. Given the small sample size, interpreting the results requires caution, and the study does not exclude the possibility of gender-specific variations in the proposed biomarkers. To ensure robustness, further investigations involving larger and more diverse samples, encompassing different genders, are necessary to validate the applicability of these biomarkers, including RHAMM, in distinguishing DKD.

## CONCLUSIONS

In summary, this study introduces a novel finding: plasma RHAMM levels can predict DKD pathogenesis in patients with T2DM. The triple complex of HA, CD44 and RHAMM, expressed on cell surfaces, shows promise as a potential targetable biomarker for managing severe renal dysfunctions.

## Supplementary Material

sfae196_Supplemental_File

## Data Availability

The data underlying this article can be shared on reasonable request to the corresponding author.
