# Supplementary material for: Elevated RHAMM as a biomarker for predicting diabetic kidney disease in patients with type 2 diabetes
Source: Clin Kidney J. 2024 Jul 3;17(7):sfae196. doi: 10.1093/ckj/sfae196 (PMC11267228; doi:10.1093/ckj/sfae196)
Supplement: sfae196_Supplemental_File [file sfae196_supplemental_file.docx]

**SUPPLEMENTAL INFORMATION:**

**Elevated RHAMM as a biomarker for predicting diabetic kidney disease in patients with type 2 diabetes**


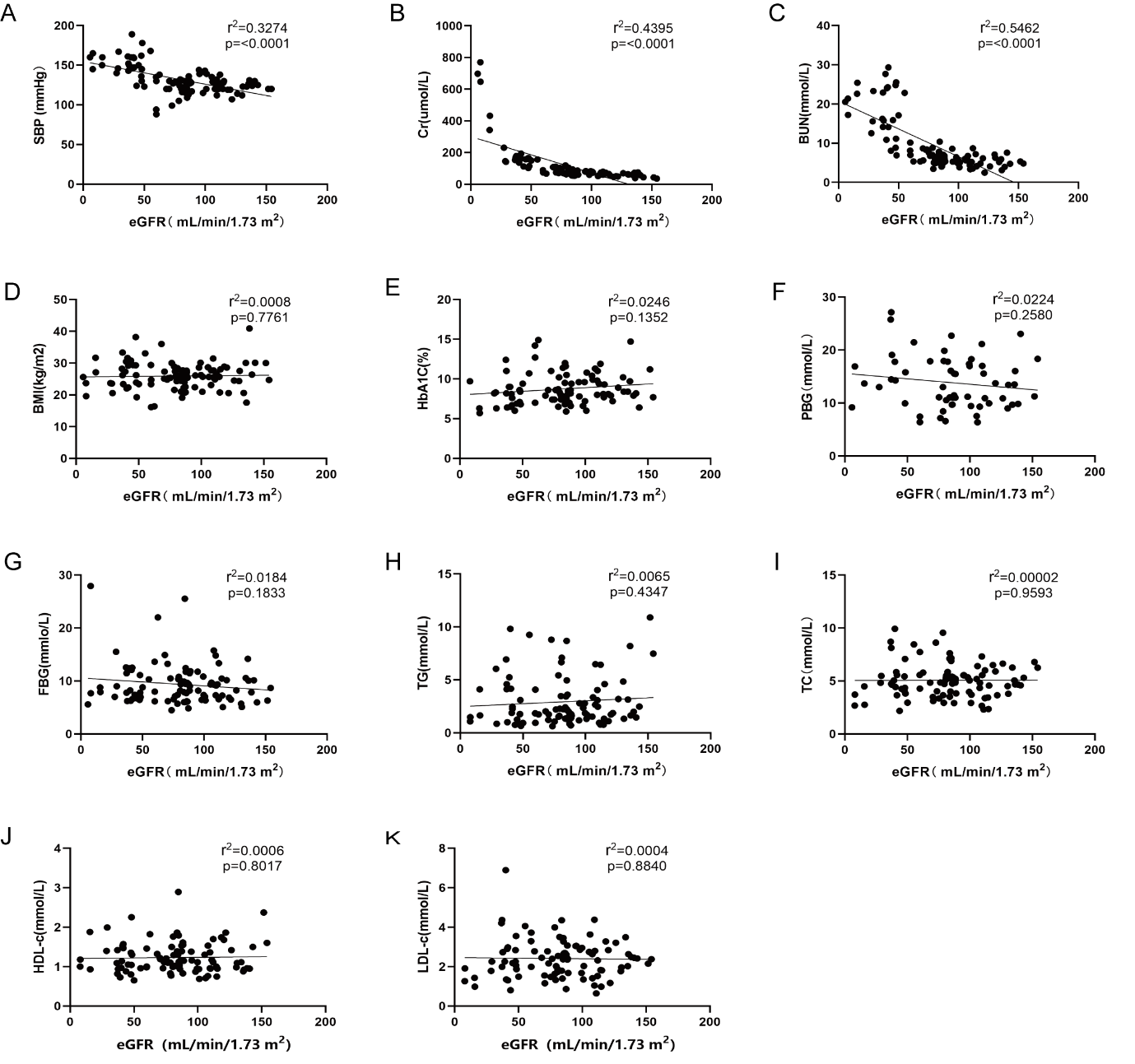


**Supplemental Figure S1 The association of eGFR with clinical parameters.** Simple linear regression was performed to evaluate the correlation between eGFR and clinical parameters. Pearson R^2^ and P values were given on the graph. Individual data points were shown with a line of best fit (solid).

**Supplemental Table S1 Comparison of clinical parameters between 3 groups.**

| Variables | | NCG  (UACR < 30) | MIG  (30≤UACR <300) | | | | | MAG  (UACR ≥ 300) | P | | |
| --- | --- | --- | --- | --- | --- | --- | --- | --- | --- | --- | --- |
| n（man/woman） | | 33（18/15） | | 31（15/16） | | | 35（16/19） | | - | | |
| Age (Years) | | 52.42±1.98 | | 61.02±1.58* | | | 58.09±2.24 | | 0.0068 | | |
| Clinical course（Years） | | 8.12±1.20 | | 9.59±1.00 | | | 16.51±1.42^*#^ | | <0.0001 | | |
| SBP（mmHg） | | 128.20±2.40 | | 139.60±2.33* | | | 149.70±2.13^*#^ | | <0.0001 | | |
| DBP（mmHg） | | 82.45±1.39 | | 86.02±1.95 | | | 84.63±1.99 | | 0.4197 | | |
| BMI（kg/m^2^） | | 24.96±0.59 | | 26.03±0.52 | | | 26.28±0.86 | | 0.3700 | | |
| TG（mmol/L） | | 2.83±0.35 | | 2.65±0.30 | | | 2.62±0.38 | | 0.9023 | | |
| TC（mmol/L） | | 5.11±0.22 | | 4.69±0.18 | | | 4.94±0.31 | | 0.3986 | | |
| LDL-c（mmol/L） | | 2.44±0.14 | | 2.22±0.11 | | | 2.35±0.21 | | 0.5405 | | |
| HDL-c（mmol/L） | | 1.24±0.06 | | 1.18±0.05 | | | 1.19±0.08 | | 0.8022 | | |
| HbA1C（%） | | 8.61±0.29 | | 8.44±0.38 | | | 8.04±0.54 | | 0.6555 | | |
| FBG（mmol/L） | | 9.99±0.86 | | 9.91±0.63 | | | 9.67±0.81 | | 0.9573 | | |
| PBG（mmol/L） | | 14.67±1.04 | | 14.04±0.73 | | | 14.16±1.41 | | 0.8911 | | |
| Cr（μmol/L） | 66.36±2.68 | | | | 71.12±2.39 | 241.70±45.88^*#^ | | | | | <0.0001 |
| BUN（μmol/L） | 5.08±0.26 | | | | 6.07±0.31 | 10.84±1.24^*#^ | | | | <0.0001 | |
| α1- microglobulin（u/L） | 22.35±0.68 | | | | 26.79±1.02 | 47.93±4.20^*#^ | | | | <0.0001 | |
| β2- microglobulin（u/L） | 1.79±0.07 | | | | 2.53±0.19 | 8.89±1.83^*#^ | | | | <0.0001 | |
| eGFR（mL/min/1.73m^2^） | 108.8±3.98 | | | | 103.7±3.82 | | | 63.29±7.86^*#^ | | | <0.0001 |

Normal Control Group (NCG: UACR<30 mg/g); Microalbuminuria Group (MIG: UACR: 30~300mg/g); Macroalbuminuria Group (MAG: UACR≥300 mg/g). ACR, Albumin-to-creatinine ratio; SBP, Systolic blood pressure; DBP, Diastolic blood pressure; BMI, Body mass index; TG, Triglyceride; TC, Total cholesterol; LDL-c, Low-density lipoprotein-c; HDL-c, High-density lipoprotein-c; FBG, Fasting blood glucose; PBG, Postprandial blood glucose; Cr, Creatinine; BUN, Blood urea nitrogen; eGFR, estimated glomerular filtration rate. **p*<0.0001 vs NCG (UACR < 30 mg/g), ^#^*p*<0.0001 vs MIG (UACR: 30~300 mg/g). Results were expressed as mean and standard error of the mean (SEM).
